# APOBEC3 inhibits DEAD-END function to regulate microRNA activity

**DOI:** 10.1186/1471-2199-14-16

**Published:** 2013-07-26

**Authors:** Sara Ali, Namrata Karki, Chitralekha Bhattacharya, Rui Zhu, Donna A MacDuff, Mark D Stenglein, April J Schumacher, Zachary L Demorest, Reuben S Harris, Angabin Matin, Sita Aggarwal

**Affiliations:** 1 Department of Genetics, University of Texas, MD Anderson Cancer Center, 1515 Holcombe Blvd, Houston, TX, 77030, USA; 2 Department of Biochemistry, Molecular Biology, and Biophysics, University of Minnesota, 321 Church Street SE, Minneapolis, MN, 55455, USA; 3 Pennington Biomedical Research Center, 6400 Perkins Road, Baton Rouge, LA, 70808, USA

**Keywords:** DND1, APOBEC3G, APOBEC3, microRNA, P27

## Abstract

The RNA binding protein DEAD-END (DND1) is one of the few proteins known to regulate microRNA (miRNA) activity at the level of miRNA-mRNA interaction. DND1 blocks miRNA interaction with the 3′-untranslated region (3′-UTR) of specific mRNAs and restores protein expression. Previously, we showed that the DNA cytosine deaminase, APOBEC3 (apolipoprotein B mRNA-editing enzyme, catalytic polypeptide like 3), interacts with DND1. APOBEC3 has been primarily studied for its role in restricting and inactivating retroviruses and retroelements. In this report, we examine the significance of DND1-APOBEC3 interaction. We found that while human DND1 inhibits miRNA-mediated inhibition of *P27*, human APOBEC3G is able to counteract this repression and restore miRNA activity. APOBEC3G, by itself, does not affect the 3′-UTR of *P27*. We found that APOBEC3G also blocks DND1 function to restore miR-372 and miR-206 inhibition through the 3′-UTRs of *LATS2* and *CX43*, respectively. In corollary experiments, we tested whether DND1 affects the viral restriction function or mutator activity of APOBEC3. We found that DND1 does not affect APOBEC3 inhibition of infectivity of exogenous retrovirus HIV (ΔVif) or retrotransposition of MusD. In addition, examination of *Ter/Ter;Apobec3−/−* mice, lead us to conclude that DND1 does not regulate the mutator activity of APOBEC3 in germ cells. In summary, our results show that APOBEC3 is able to modulate DND1 function to regulate miRNA mediated translational regulation in cells but DND1 does not affect known APOBEC3 function.

## Background

The RNA binding protein DEAD-END (DND1) is essential for germ cell viability [1,2]. When *Dnd1* is functionally inactivated, as in the *Ter* mutant mouse strain, this results in death of germ cells, sterility [2], and in some cases development of testicular germ cell tumors [2,3].

DND1 encodes canonical RNA recognition motifs [1,4] through which it interacts with the 3′-UTRs of mRNAs. For example, DND1 inhibits miR-221 function from the 3′-UTR of *P27* resulting in increased P27 protein expression [4,5]. Two U-rich DND1 binding sites have been mapped adjacent to two miR-221 binding sites in the 3′-UTR of *P27*[4]. DND1 has also been shown to inhibit miR-372 from the 3′-UTRs of *LATS2* (serine/threonine-protein kinase, large tumor suppressor, homolog 2) and inhibit miR-1 and miR-206 from the 3′-UTRs of *CX43* (connexin-43) [4]. However, DND1 binding sites have not been mapped within the 3′-UTRs of *LATS2* or *CX43*.

miRNA association with mRNA usually results in translation inhibition or degradation of mRNA. It is thought that DND1 binds to mRNA and prevents miRNAs and miRISC (miRNA-induced silencing complexes) from binding. miRISCs are composed of ribonucleoproteins that assemble with the miRNA and mediate either translational repression or degradation of mRNA [6-8]. Alternately, DND1 may bind and sequester mRNAs away from miRNA access.

Although DND1 was initially identified for its role in germ cells and germ cell tumors, emerging evidence indicates a wider role for DND1 in mammalian tissues, especially in cancers. For example, over-expression of DND1 is detected in some histological sub-types of human testicular cancers, leukemia, lung and ovarian cancers (ONCOMINE and NCBI Geo Profiles). A recent study detected DND1 in human tongue squamous cell carcinoma (TSCC) and found that miR-24 directly targets *DND1* mRNA [9]. Up-regulation of miR-24 decreased DND1 expression resulting in lower P27 levels and increased proliferation and reduced apoptosis in TSCC cells. Another study showed that *Ras* transformed keratinocytes down regulate DND1 which results in increased miR-21 mediated inhibition of MSH2 [10].

Work in our laboratory and others show that DND1 interacts with a broad range of mRNA targets [11,12]. The targets include transcripts encoding cell cycle regulators (*P27, TP53, LATS2*), pluripotency factors (*OCT4, SOX2, NANOG*) and pro- and anti-apoptotic factors (*BAX* and *BCLX*). Expression of these genes is required at specific developmental stages in germ cells such as during active proliferation or quiescence. Because DND1 interacts with a range of mRNAs, this raises the question as to the factors which might serve to modulate DND1 interaction with physiologically appropriate targets. Therefore one important goal is to determine how DND1 function is regulated in cells.

In a related study, we found that DND1 interacts with APOBEC3 [13]. We showed that mouse DND1 immunoprecipitated with mouse APOBEC3 in mammalian cells, including in germ cells. In addition, fluorescent tagged DND1 and APOBEC3 co-localized at peri-nuclear regions in mammalian cells.

One well-studied function of mouse Apobec3 and its human counterpart, APOBEC3G (apolipoprotein B mRNA-editing enzyme, catalytic polypeptide-like 3G, A3G) is contributing to innate immunity through retrovirus and retrotransposon restriction [14-16]. Restriction occurs through a well-established cDNA cytosine deamination mechanism and by a less well-characterized deamination-independent mechanism [17-20]. Human APOBEC3G and mouse APOBEC3, each possess two zinc-binding motifs [21,22]. The active domain is responsible for deaminase activity and the pseudo-active domain contributes most of the RNA/ssDNA binding affinity [23-27]. Although both human and mouse proteins have such a division of labor, the domain organization is opposite with N-terminal of mouse APOBEC3 and the C-terminal domain of human APOBEC3G active for deamination [28-30].

Other studies indicate that overexpression of specific members of the human APOBEC3 family (such as the single cytidine deaminase domain containing, APOBEC3A) can hypermutate the cellular genome or mitochondrial DNA [31,32]. Thus APOBEC3 family members are potentially powerful mutators [33] and very likely cells possess mechanisms to keep the latent deleterious activity of APOBEC3 in check. One way that cells protect their genomes from APOBEC3 is that mouse APOBEC3 and most human APOBEC3 proteins are localized to the cytoplasm [14,29,34].

In this report, we examined the significance of APOBEC3 interaction with DND1. Our results show that APOBEC3 can oppose DND1 function to restore miRNA-mediated inhibition of translation. We therefore propose that interaction of APOBEC3 with DND1 may be one way in which DND1 activity is regulated in cells.

## Methods

### Transient transfections

Human DND1 with HA tag in C-terminus was cloned into pCDNA3.1 nV5-DEST (Invitrogen) expression vector (DND1-HA). Human APOBEC3G with myc tag in C-terminus was cloned into pcDNA3.1(+)(APOBEC3G-myc). Transient transfections were performed using the 293 T cell line as this cell line has previously been used for testing DND1 function [4] and the results using 293 T were similar to that using other cell lines such as MCF-7 and Tera1. On advantage is that 293 T cells take up transfected DNA efficiently to give reproducible results. 293 T cells were cultured in DMEM supplemented with 10% fetal bovine serum in 5% CO_2_ at 37°C. The cells were transiently transfected using SuperFect transfection reagent (QIAGEN) with 1 ng pGL3-P27-3′UTR [4] together with constructs encoding miRVec-221 (50 ng), DND1-HA (10 ng) and/or APOBEC3G-myc (1 ng to 25 ng range). *LacZ* expression constructs (4 ng) were co-transfected into all cells. Equivalent amounts of DNA were introduced into all cells with pGEM DNA being used to equalize for DNA levels used for transfections. After 48 h the cells were washed and treated with cell culture lysis buffer (Promega). 5 uL of the lysates were used for luciferin assays. All transfection experiments were performed in triplicates. Results shown are the mean and standard error from three independent experiments. Similar transfections also tested the effect of DND1, APOBEC3G and miR-372 (mirVec-372) on pGL3 3′UTR LATS2, and miR-206 (miR vec-206) on pGL3 Cx43 3′UTR and pGL3-control vector. Mutant P27 vectors used were pGL3 3′UTR min mut1 (m1 or mut1, in which both DND1 binding sites are mutated) and pGL3-p27mut-3′-UTR (m3; in which both miRNA binding sites are mutated) [4].

### Statistical analysis

Data are expressed as mean ± standard deviation/or standard error. Statistical analyses were performed using GraphPad Prism (software version 5.0. VA). Differences were determined by Student’s t test. A *P* value of < 0.05 was considered significant.

### Luciferase assays

The assays were performed using Luciferase assay kit (Pomega) according to manufacturer’s directions. β-galactosidase assay results were used to normalize the transfection efficiencies. β-galactosidase assays were done using beta-Glo assay kit (Promega) according to manufacturer’s direction.

### Immunoblotting

DND1-HA and APOBEC3G-myc expression in transfected cells was detected in cell lysates using anti-myc and anti-HA antibodies, as described [13].

### Viral infectivity and MusD transposition assays

Single cycle infectivity assays for HIV(ΔVif) were performed using 293 T cells as described in [28]. MusD transposition assay in HeLa cells were performed as described in [35]. Expression vectors encoding mouse DND1 and APOBEC3 have been described [13,36].

### Mouse crosses

*Ter/+* mice were intercrossed with *Apobec3*−/− mice. (Both copies of wild-type DND1 are functionally inactivated in *Ter/Ter* mice.) The F1 mice were selected by genotyping and *Ter/+;Apobec3+/−* mice were intercrossed to generate *Ter/Ter;Apobec3−/−* mice [2,37]. The F2 mice were genotyped and the testes of double homozygote mice were examined for presence of germ cells. All mice were housed in the standard mouse Plexiglas cages in a room maintained at constant temperature and humidity under a 12 h light and darkness cycle. Animals were fed irradiated pelleted chow and water ad libitum. The experimental protocol was reviewed and approved by the Institutional Animal Care and Use Committee at MD Anderson Cancer Center.

## Results

### APOBEC3 inhibits DND1 function

**Figure 1:**
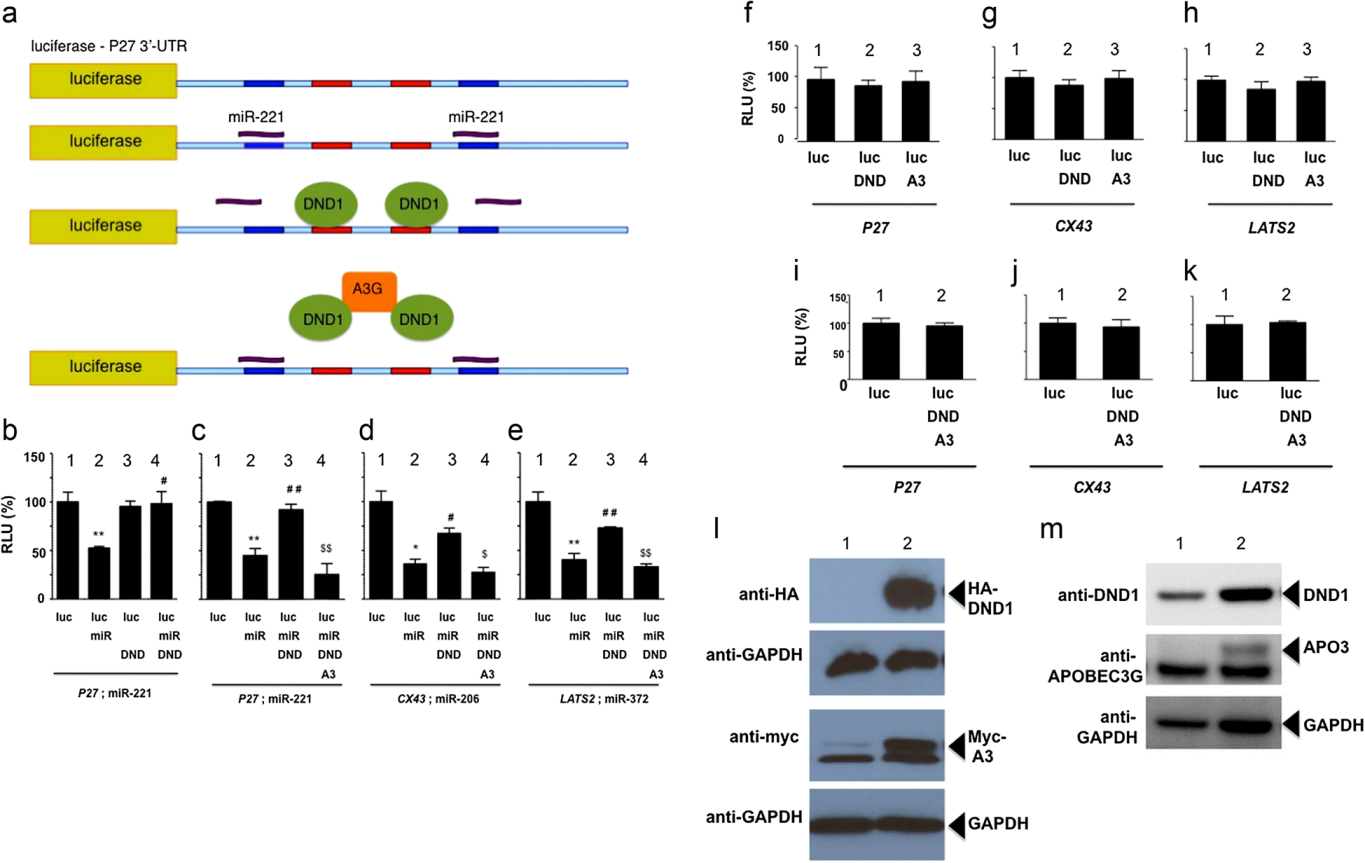
**APOBEC3G (A3) inhibits DND1 function. (a)** Diagram of luciferase construct pGL3-P27-3’-UTR showing miR-221 (blue) and DND1 binding sites (red) [4]. **(b)** DND1 blocks the effect of miR-221. Luciferase activity of pGL3-P27-3’UTR (or luc-*P27* ) alone (lane 1); in the presence of miR-221 (lane 2); in the presence of DND1 (lane 3); in the presence of miR-221 and DND1 (lane 4). (**) miR-221 inhibited luc-*P27* expression (*P* = 0.005) and (#) DND1 rescued the inhibition (*P* = 0.011). *LacZ* constructs were co-transfected into cells and β-galactosidase used for normalization of transfection efficiencies. Results are the mean and standard error from 3 independent experiments. (RLU = relative luminescence unit is the ratio between luciferase and β-galactosidase level, adjusted to 100%). Vectors encoding 1 ng luciferase, 50 ng miR, 10 ng DND1 and 10 ng APOBEC3G were used in all assays. **(c)** APOBEC3G counteracts the effect of DND1. Luciferase activity of pGL3-P27-3’UTR in presence of miR-221, DND1 and APOBEC3G (lane 4). (**) miR-221 inhibited luc-*P27* (*P*= 0.004) (lane 2), (##) DND1 rescued the inhibition (*P*= 0.008) (lane 3) whereas ($$) APOBEC3G (A3) opposed the effect of DND1 (*P* = 0.009) (lane 4). Differences between (**) lane 2 and ($$) 4 are not statistically significant (*P*=0.0859). **(d)** pGL3-Cx43-3’UTR (Luciferase-CX43-3’UTR or luc-*CX43*) was cotransfected with miR-206, DND1 and APOBEC3G. (*) miR-206 inhibited luc-*CX43* (*P*= 0.017) (lane 2), (#) DND rescued the inhibition (*P*= 0.024) (lane 3) whereas ($) A3 opposes DND1(*P* =0.016) (lane 4). Differences between (*) lane 2 and ($) 4 are not statistically significant (*P*= 0.17). **(e)** pGL3-3’UTR-LATS2 (Luciferase-LATS2-3’UTR or luc-*LATS2*) was cotransfected with miR-372, DND1 and APOBEC3G. (**) miR-372 inhibited luc-*LATS2* (*P*= 0.001) (lane 2), (##) DND rescued the inhibition (*P*= 0.001) (lane 3), ($$) whereas A3 opposes DND1 (*P* = 0.001) (lane 4). Differences between (**) lane 2 and ($$) 4 are not statistically significant (*P*=0.176). **(f)** Control experiments demonstrate no significant effect of DND1 (lane 2) (*P* = 0.2860) or APOBEC3G alone (lane 3) (*P* =0.4356) on pGL3-p27-3’UTR; **(g)** no significant effect of DND1 (lane 2) (*P* = 0.1426) or APOBEC3G alone (lane 3) (*P* = 0.4196) on pGL3-Cx43-3’UTR; **(h)** no significant effect of DND1 (lane 2) (*P* = 0.1779) or APOBEC3G alone (lane 3) (*P* = 0.4615) on pGL3-3’UTR-LATS2. **(i)** Control experiments demonstrate no significant effect when DND1 together with APOBEC3G (lane 2) are transfected with pGL3-p27-3’UTR (*P* = 0.3433), **(j)** or pGL3-Cx43-3’UTR (*P*= 0.3565), **(k)** or pGL3-3’UTR-LATS2 (*P* = 0.4183). **(l)** Expression of HA-tagged DND1 (arrow) and myc-tagged APOBEC3G (myc-A3) (arrow) in 293T cells (lane 1) and 293T transfected with DND1-HA and APOBEC3G-myc (lane 2). Immunoblotting was using anti-HA, anti-myc and anti-GAPDH. **(m)** Expression of endogenous DND1 (arrow) and APOBEC3G (arrow) in 293T cells (lane 1) and 293T transfected with DND1-HA and APOBEC3G-myc (lane 2). Immunoblotting was using anti-DND1, anti-APOBEC3G and anti-GAPDH antibodies.

Kedde *et. al*. [4] showed that DND1 blocks miR-221 function from the 3′-UTR of *P27* mRNA. Also, a report by Huang *et. al*. [38] found that human APOBEC3G can inhibit miRNA function from mRNAs and this indicated a novel role of APOBEC3G in regulating miRNA activity and protein translation. Therefore, we asked whether APOBEC3 affects this function of DND1 and tested the effect of human APOBEC3G on DND1. Because 3′-UTR of human *P27* has been characterized to contain two DND1 binding sites flanked by miR-221 binding sites (Figure 1a) [4], constructs encoding human *P27* were predominantly used in these assays, together with human DND1 and APOBEC3G. We used the reporter construct, pGL3-P27-3′UTR [4], in which the 3′-UTR of human *P27* has been cloned downstream to luciferase reporter gene (Figure 1a). pGL3-P27-3′UTR was co-transfected into 293 T cells together with expression vectors encoding miR-221, HA-tagged DND1 (HA-tag in C-terminus of DND1) and myc-tagged APOBEC3G (myc-tag in C-terminus of APOBEC3G). Luciferase assays were carried out to monitor the effect of APOBEC3G and DND1 on miR-221 activity on *P27* 3′-UTR.

As expected, we found that miR-221 inhibits pGL3-P27-3′UTR luciferase activity (*P* = 0.005). DND1 counteracts the effect of miR-221 to restore luciferase activity (*P* = 0.01) (Figure 1b). However, when we introduce APOBEC3G together with DND1, we found that presence of APOBEC3G opposes DND1 repression of miR-221 (*P* = 0.009) (Figure 1c). Thus APOBEC3G restores miR-221 inhibition of pGL3-p27-3′UTR luciferase activity.

APOBEC3G had a similar effect of blocking DND1 function to restore miR-206 inhibition from *CX43* (connexin-43; pGL3 Cx43 3′UTR) (*P* = 0.02) and to restore miR-372 inhibition from the 3′-UTR of *LATS2* (pGL3 3′UTR LATS2) (*P* = 0.001) (Figure 1d and 1e). In these experiments, APOBEC3G restored the miR-mediated inhibition of luciferase activity but did not further increase inhibition. We conclude therefore, that APOBEC3G may have a general function in blocking DND1 activity. We verified that transfected plasmids encoding HA-tagged DND1 and myc-tagged APOBEC3G were expressed in the 293 T cells (Figure 1l). We also detected expression of endogenous DND1, but not APOBEC3G, in 293 T cells (Figure 1m).

Control experiments demonstrated no significant effect of either DND1 or APOBEC3G alone (Figure 1f-h) or DND1 plus APOBEC3G on luciferase activity of pGL3-P27-3′-UTR, pGL3-Cx43-3′UTR or pGL3-3′UTR-LATS2 (Figure 1i-k).

**Figure 2:**
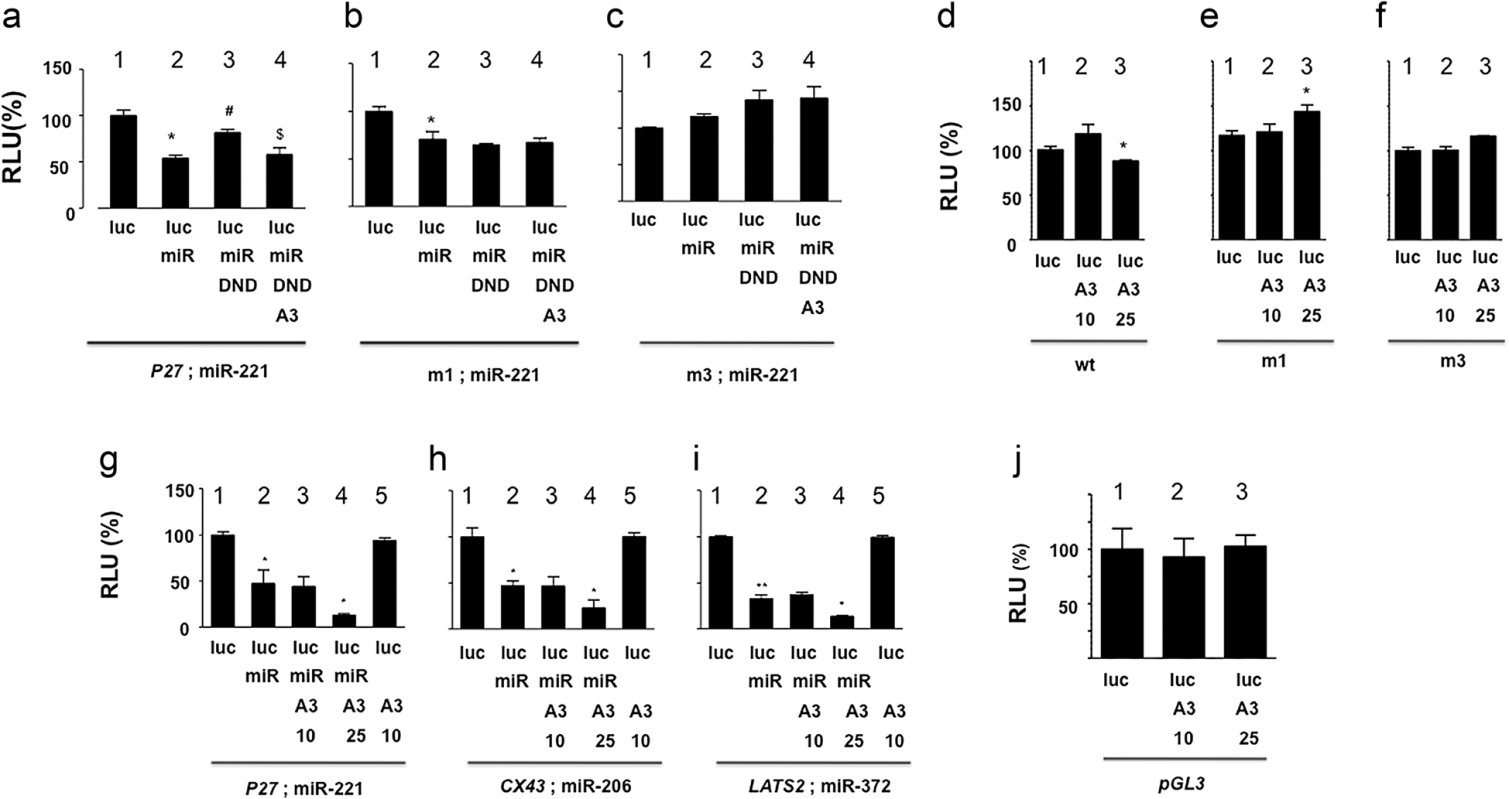
**APOBEC3G likely functions through DND1 to restore miRNA activity. (a)** APOBEC3G blocks DND1 to restore miR-221 inhibition of luciferase from wild-type *P27*-3’-UTR (lane 4). (*) miR-221 inhibited luc-P27 expression (*P* = 0.0116) (lane 2), (#) DND1 rescued the inhibition (*P*= 0.0138) (lane 3). ($) APOBEC3G opposed the function of DND (*P* = 0.0467) (lane 4). **(b)** APOBEC3G does not restore miR-221 inhibition from mutated *P27* 3’-UTR in m1 (or luc-m1; in which both DND1 binding sites mutated) [4] (lane 4). (*) miR-221 inhibited luc-m1 expression (*P*= 0.0460). DND1 does not rescue miR-221inhibition (*P*=0.2665) (lane 3) and A3 does not affect DND1 function (*P* = 0.3211). **(c)** miR-221 does not inhibit m3 (or luc-m3, both miRNA binding sites mutated [4]) (*P*= 0.2665) (lane 2). DND1 (P= 0.1184) (lane 3) or A3 (P= 0.4569) (lane 4) also do not affect luc-m3 activity. **(d)** Effect of increasing levels of APOBEC3G on wild-type pGL3-P27-3’-UTR. APOBEC3G was cotransfected at two concentrations (10 ng and 25 ng). Higher APOBEC3G (A3) (25 ng) inhibits (*) pGL3-P27-3’-UTR (lane 3) (*P*=0.023). **(e)** Higher A3 (25 ng) slightly enhances luc-m1 expression (*) (lane 3) (both DND1 binding sites mutated) (*P*=0.0432). **(f)** Higher A3 (25 ng) also enhances luc-m3 expression (*) (lane 3) (both miR-221 binding sites mutated) (*P* = 0.0141). However, lower A3 levels (10 ng) have no effect on pGL3-P27-3’-UTR, luc-m1, luc-m3 expression (lane 2 in **d**, **e** and **f**: *P*= 0.0798, 0.3567 and 0.4544 respectively). **(g)** Effect of increasing APOBEC3G on miR function. miR-221 inhibits *luc-P27* (*P* = 0.0153) (lane 2). A3 (10 ng), together with miR-221, does not further inhibit *luc-P27* (*P* = 0.4553). However, A3 at 25 ng (lane 4), together with miR-221, further inhibits *luc-P27* (*P*= 0.0488). **(h)** miR-206 inhibits luc-*CX43* (*P*=0.0105) (lane 2). A3 (10 ng), together with miR-221 does not further inhibit *luc-CX43* (*P* = 0.4765). However, 25 ng A3 (*) (lane 4), together with miR-221, further inhibits luc-*CX43* (*P*= 0.0385). **(i)** miR-372 inhibits luc-*LATS2* (*P*= 0.0011) (lane 2). A3 (10 ng), together with miR-221 does not further inhibit luc-*LATS2* (*P*= 0.1673). However, 25 ng A3 (*) (lane 4), together with miR-221, further inhibits luc-*LATS2 (P* = 0.013). **(j)** Increasing levels of APOBEC3G have no effect on luciferase translation. Control luciferase reporter, pGL3-luciferase (1 ng) was cotranfected with increasing concentrations (10 ng and 25 ng) of APOBEC3G expression constructs (*P*= 0.4021 and *P* = 0.4573 respectively).

In further control experiments, we used *P27* 3′-UTR constructs in which the DND1 or miRNA binding sites were mutated [4] (Figure 2b and c). When both DND1 binding sites on pGL3-P27-3′-UTR were inactivated (m1-luc), miR-221 was able to suppress luciferase activity but DND1 was unable to rescue miR-221 inhibition (*P* = 0.3) and APOBEC3G was not able to restore miR-221 inhibition (*P* = 0.3) (Figure 2b). This suggests that APOBEC3G likely functions, at least partially, through its interaction with DND1 or through DND1 binding sites on *P27*. Use of mutant *P27* 3′-UTR constructs in which the miRNA binding sites were mutated (m3-luc) [4] (Figure 2c) showed, as expected, no functional inhibitory effect of mir-221 or any further effects by DND1 or APOBEC3G.

Next we tested the effect of higher APOBEC3G concentrations on normal and mutant luciferase expression constructs (Figure 2d-i). Levels that we normally use in our reporter assays (10 ng), had no effect on pGL3-P27-3′-UTR, luc-m1, luc-m3 expression (Figure 2d-f). However, higher levels of APOBEC3G (25 ng) slightly inhibited pGL3-P27-3′-UTR (*P* = 0.023) expression and on the other hand, slightly enhanced luc-m1 (both DND1 binding sites mutated) (*P* = 0.04) and luc-m3 expression (both miR-221 binding sites mutated) (*P* = 0.01) (Figure 2d-f). We don’t fully understand why higher levels of APOBEC3G affect luc-P27, m1 and m3 activity. One possibility is that at higher concentrations, APOBEC3G may have additional functions such as ability to interact with the 3′-UTRs to inhibit translation and luciferase activity (of wild-type P27) or promote translation (from mutant m1 and m3).

Higher APOBEC3G, when present together with miR-221, also further inhibited pGL3-P27-3′-UTR (or luc*-P27)* (*P* = 0.0488), luc-*CX43* (*P* = 0.0385) and luc-*LATS2 (P* = 0.013) (Figure 2g-i). One explanation for this is that because 293 T cells express endogenous DND1 (Figure 1m), higher concentrations of APOBEC3G are able to more effectively block the effect of both endogenous and transfected DND1 to restore miRNA-mediated inhibition. Also, interestingly, an earlier study showed that APOBEC3G can, by itself, inhibit miRNA activity, and these studies utilized higher, microgram levels of APOBEC3G [38].

We also tested the direct effect of APOBEC3G on pGL3-control vector. Increasing levels of APOBEC3G did not affect the luciferase activity from pGL3-control vector implying that APOBEC3G did not affect translation of luciferase (Figure 2j). The pGL3 vector does not have an extensive 3′-UTR with miRNA or DND1 binding sites unlike the 3′-UTRs of *P27*, *CX43* or *LATS2,* and therefore is not affected by higher levels of APOBEC3G.

In summary, these experiments lead us to conclude that APOBEC3G is able to block DND1 function and restore miRNA-mediated translation repression. APOBEC3G does not directly affect the 3′-UTRs of *P27, CX43* or *LATS2* or miRNA interaction with mRNAs. However, higher levels of APOBEC3G appear to have additional independent functions on the 3′-UTRs of genes.

### DND1 does not affect APOBEC3 function

**Figure 3:**
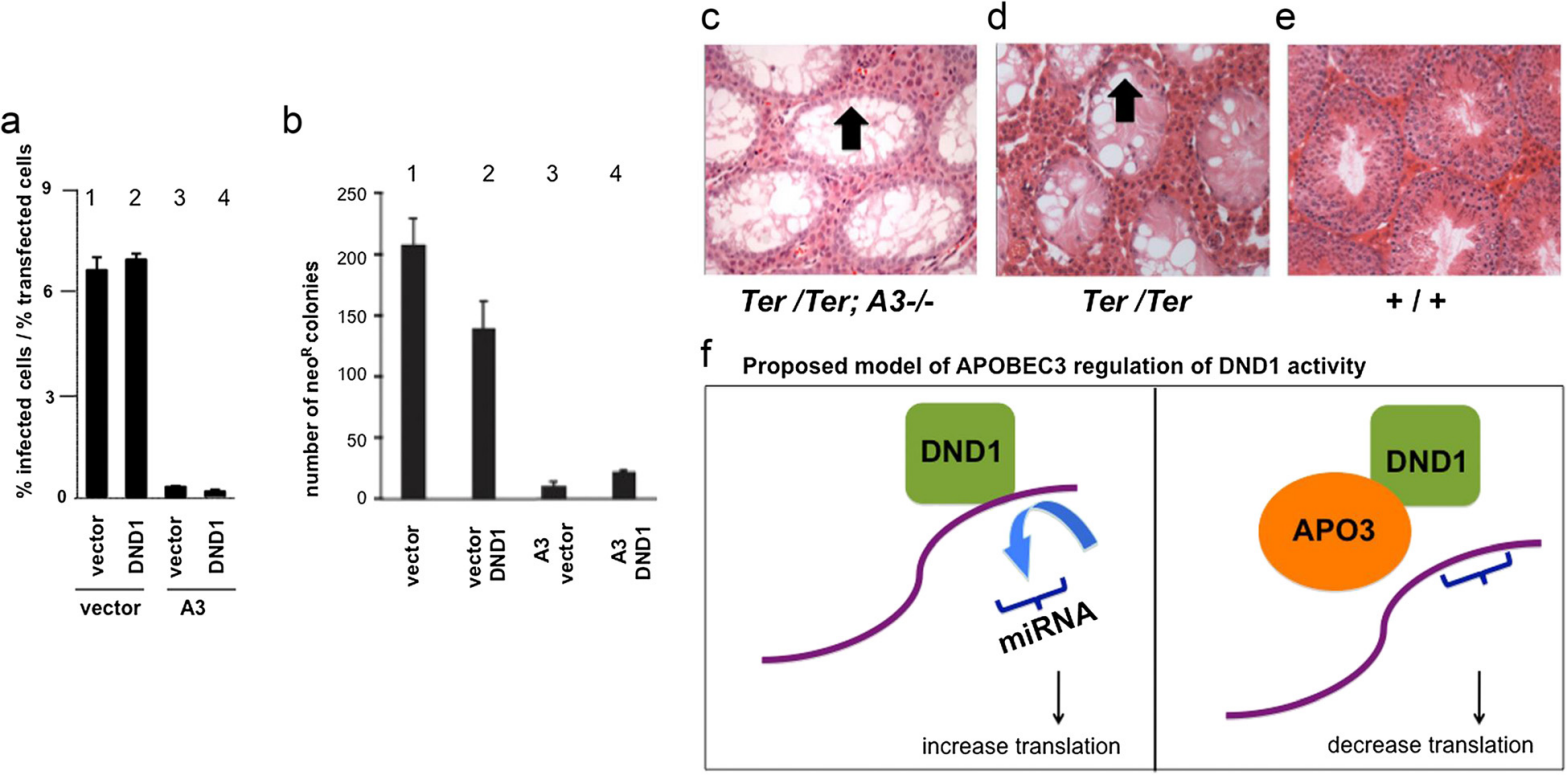
**DND1 does not affect APOBEC3 function. (a)** DND1 does not affect APOBEC3 antiretroviral activity. Infectivity of HIV-GFP produced in the presence of control vector (vector) (lane 1) or vector encoding mouse DND1 (lane 2) (*P* = 0.2082). Infectivity of HIV-GFP produced in the presence of mouse APOBEC3 (A3) (lane 3) and APOBEC3 plus DND1 (lane 4) (*P* = 0.1464). Empty vector was used to equalize the amount of construct used in each assay lane. Results from two independent experiments were averaged. Error bars indicate the difference in infectivity observed between the two experiments. **(b)** DND1 does not affect MusD restriction by APOBEC3. Effect of control vector (lane 1), mouse DND1 (lane 2), mouse APOBEC3 (lane 3), both mouse DND1 and APOBEC3 (lane 4) on MusD retrotransposition, relative to the vector control. Transposition was monitored by the number of G418-resistant colonies. Results from two independent experiments were averaged. *P* value comparing lanes 3 and 4 is 0.10980. **(c)** Histology section through testes of double homozygous male *Ter/Ter ;A3−/− (or Ter/Ter; Apobec3−/−),***(d)***Ter/Ter* and **(e)** wild-type (+/+) mice. Arrow points to lumen in seminiferous tubules showing lack of germ cells persist in *Ter/Ter ; A3−/−* testis similar to that in testis of *Ter/Ter* mice. Testes of *Apobec3−/− (A3−/−)* mice have normal wild-type germ cell histology similar to +/+ (not shown). **(f)** Proposed model how APOBEC3G regulates DND1 function. DND1 blocks miRNA activity (left panel). We observe decreased protein translation (as measured by luciferase activity) in the presence of APOBECG3 suggesting that APOBEC3G blocks DND1 function. When DND1 binding sites on P27-3′-UTR are inactivated, both DND1 and APOBEC3G fail to affect luciferase activity. This suggests that APOBEC3G functions through DND1, may be by removing or sequestering DND1 to restore miRNA access to the 3′-UTR of *P27* (right panel).

The above results show that APOBEC3G can block DND1 function to regulate miRNA activity. However, mouse APOBEC3 and human APOBEC3G have been widely studied for their role in inhibiting viral infectivity [14-16]. Therefore, we tested whether DND1 affects APOBEC3 function of inhibiting retroviral infectivity [28,39]. Single cycle infectivity assay shows that, as expected, mouse APOBEC3 severely reduces HIV(ΔVif) viral infectivity in 293 T cells (Figure 3a, lane 3). However, presence of mouse DND1 together with APOBEC3 did not change ability of APOBEC3 to reduce HIV(ΔVif) infectivity (Figure 3a, lane 4). DND1 alone also had no effect on HIV(ΔVif) viral infectivity (Figure 3a, lane 2). Empty vector was used to equalize the amount of construct used in each assay lane [28,39].

In a second set of experiments, we tested whether DND1 affects the ability of APOBEC3 to inhibit MusD retrotransposition [35]. APOBEC3, by itself, drastically inhibits MusD retrotransposition (as indicated by decrease in neo^R^ colonies by Apo3 + vector) (Figure 3b, lane 3). However, DND1, when combined with APOBEC3, did not significantly affect APOBEC3 function of inhibiting MusD retrotransposition (A3 + DND1, lane 4). DND1, by itself, also had no effect on MusD retrotransposition (vector + DND1, lane 2). The results from these two experiments indicate that DND1 does not modulate the viral restriction function of APOBEC3.

To further explore the functional relationship between *Dnd1* and *Apobec3*, we asked whether the germ cell phenotype of mice lacking wild-type *Dnd1* (*Ter* mutant mice) is dependent on *Apobec3*. It is known that APOBEC3 family members are potentially powerful mutators [31-33]. We reasoned that interaction of DND1 with APOBEC3 in germ cells may be one mechanism to keep the latent deleterious activity of APOBEC3 in check. Thus it is formally possible that depletion of germ cells in mice lacking normal *Dnd1* (as observed in *Ter* mutant mice) is due to uncontrolled Apobec3 genomic mutating activity. We therefore tested whether removing *Apobec3* from mice in which *Dnd1* is inactivated, would restore normal germ cells. Mice lacking wild-type *Dnd1* (in *Ter* mice) have no germ cells [2] but mice lacking *Apobec3* are normal and fertile [37]. Thus, we generated double mutant mice lacking both wild-type *Dnd1* and *Apobec3* (*Ter/Ter*;*Apobec3*−/− or *Ter/Ter*;*A3*−/−) mice. However, examination of newborn or adult testes of *Ter/Ter*;*A3*−/− mice indicated that they have no germ cells and are sterile (Figure 3c) and are indistinguishable from *Ter/Ter*;*A3+/+* animals (Figure 3d). Thus this genetic assay suggests that DND1 does not regulate the mutator function of APOBEC3 in germ cells and germ cell loss.

in *Ter/Ter* mice is likely not due to unregulated activity of APOBEC3. Together, results from the restriction assays and the genetic crosses are consistent with the idea that DND1 does not modulate mouse APOBEC3 activity.

## Discussion

Our results demonstrate that APOBEC3G is able to block DND1 function and restore miRNA mediated inhibition of translational repression. This function of APOBEC3G appears to apply to multiple mRNA targets of DND1 as APOBEC3G has a similar effect on *P27*, *LATS2* and *CX43*. On the other hand, DND1 does not appear to affect the viral restriction function of APOBEC3. In addition, our genetic crosses suggest that DND1 interaction with APOBEC3 does not regulate the mutator function of APOBEC3 in germ cells.

The mechanism of how APOBEC3G blocks DND1 remains to be determined. At least, three possible mechanisms can be proposed. The first possibility is that APOBEC3G may bind to DND1 and sequester it away from mRNAs and miRNAs (Figure 3f). In support of this, we have observed that mouse APOBEC3 and DND1 co-immunoprecipitate and co-localize in cells [13].

The second possibility is that APOBEC3G may bind mRNAs (maybe together with DND1) and subsequently interact with components of the miRISC to activate translation repression or interact with translation initiation factors to inhibit them. Indeed, mass spectrometric analysis show that a large number of cellular RNA-binding proteins associate with APOBEC3G [40-42]. Some of these are known components of the miRISC such as ARGONAUTE 1(Ago1), ARGONAUTE 2 (Ago2), GW182, MOV10, YB-1, DCP1A and RCK/P54, and are involved in post-transcriptional silencing of gene expression [38,41-43]. These interactions of APOBEC3G with RNA binding proteins were found to be either direct protein-protein interactions or mediated by RNA. In addition, confocal microscopy experiments showed that APOBEC3G co-localized with many of the miRISC RNA-binding proteins to mRNA processing, P-bodies [40,41]. Thus it is conceivable that interaction of APOBEC3 with specific miRISC proteins may override the effect of DND1 to enhance miRNA activity.

The third possibility is that the cytidine deaminase activity of APOBEC3 may allow it to edit the 3′-UTR sequences of *P27*, *LATS2* and *CX43* to inhibit DND1 binding. Interestingly, indirect support of this hypothesis comes from recent reports by a number of groups that analysed deep sequencing data and found greater than expected incidence of editing present in the mammalian transcriptome [44,45]. As to which of the three possible mechanisms apply to APOBEC3G blocking DND1 function is currently under investigation.

APOBEC3 proteins have been studied as factors that restrict viruses and retrotransposons. However, we found that DND1 has no effect on the viral restriction function of APOBEC3. APOBEC3G can bind both cellular RNAs and RNA binding proteins [27,40,41] and the RNA binding activity of the N-terminal cytidine deaminase domain of APOBEC3G is essential for viral restriction [23,24]. The significance of APOBEC3G interactions with cellular proteins and RNAs is not clear. However, during viral infection of cells, such as HIV-1 (Vif) infection of T lymphocytes, APOBEC3G gains access to viral particles through a ribonucleoprotein interaction and thus APOBEC3 binding to RNA is a critical for antiviral function [23]. The incorporation of APOBEC3G into new viral particles allows it to be released into infected cells where APOBEC3G can deaminate the replicating viral cDNAs to effect reduction of viral infectivity. Interestingly, it has been shown that other P-body proteins, such as MOV10 [46-48] are also involved in reducing the infectivity of exogenous retroviruses and retrotransposons. Thus, P-body protein components such as MOV10, and as we report here, APOBEC3G, participate both in miRNA silencing and viral restriction processes.

An earlier study on APOBEC3G function showed that APOBEC3G can, by itself, inhibit miRNA activity [38]. The previous study used luciferase constructs encoding only miRNA binding sites or encoding multiple miRNA binding sites in tandem. In contrast, our assays used the 3′-UTR of endogenous genes to test APOBEC3G activity and we also tested how APOBEC3G blocks DND1 function on these 3′-UTRs. Moreover, the amount of APOBEC3G expression vectors transfected into cells were considerably higher in the study [38] and we also found that higher APOBEC3G levels may have alternate effects on the 3′-UTRs and miRNA function. Another possibility is that APOBEC3G may have different effects on different miRNAs and transcripts, inhibiting some miRNAs while activating others.

One caveat of our studies is that the miRNA studies were performed using human DND1 and APOBEC3G whereas the infectivity assays and genetic studies were performed using mouse factors. We did this because human DND1 function was previously characterized using human *P27* 3′-UTR and DND1 binding sites have been mapped in the 3′UTR of *P27*[4]. Humans encode multiple members of the APOBEC3 family proteins (APOBEC3A, 3B, 3C, etc.) [21]. We selected human APOBEC3G for our miRNA studies which is functionally most closely related to mouse APOBEC3. The role of the other human APOBEC3 factors in inhibiting DND1 activity remains to be determined.

APOBEC3 proteins are expressed in germ cells [13,21,49]. But although APOBEC3 proteins inhibit retrotransposition [17,50,51], to date, this has not been demonstrated in germ cells. In fact, *Apobec3* null mice are normal and fertile [37]. We found that double null *Ter/Ter*;*Apobec3*−/− mice have similar phenotype as *Ter/Ter* mice and lack of *Apobec3* does not rescue the *Ter/Ter* (*Dnd1−/−)* phenotype to restore germ cells. This supports the idea that DND1 does not regulate the mutator activity of APOBEC3 in germ cells, and deregulated APOBEC3 mutator activity is not responsible for germ cell loss in *Ter/Ter* (*Dnd1−/−)* mice.

In summary, our studies focus on a novel aspect of APOBEC3 function in that we show APOBEC3G regulates DND1 function and in this way affects miRNA activity. Viral restriction and miRNA mediated gene silencing are evolutionarily related processes utilizing similar protein complexes, which localizes to cytoplasmic RNA granules. Our data provides compelling evidence that APOBEC3G may be involved in both these processes.

## Conclusion

We present our novel finding that RNA binding protein DEAD END (DND1) blocks miRNA function to permit translation, and APOBEC3G (apolipoprotein B mRNA-editing enzyme, catalytic polypeptide like 3) antagonizes DND1 to reduce translation. Not much is known about how microRNA target interactions are regulated by RNA binding proteins, thus our result advances an area of importance to help understand how microRNA activity can be modulated in response to signals.

## Abbreviations

3′-UTR: 3′-untranslated region; Mouse APOBEC3 and human APOBEC3G: Apolipoprotein B mRNA-editing enzyme, catalytic polypeptide like 3; LATS2: Serine/threonine-protein kinase, large tumor suppressor, homolog 2; CX43: Connexin-43; miRNA: microRNA; mRNA: Messenger RNA; P27, LATS2: Human genes and transcripts are in italics and capitals; P27, LATS2, APOBEC3G: Human and mouse proteins are in capitals; Apobec3: Mouse genes and transcripts are in italics and lowercase.

## Competing interests

The authors declare that they have no competing interests.

## Authors’ contributions

AM and SA conceived and designed the experiments, performed analysis and interpretation of data and wrote the manuscript. NK performed the transfection assays. CB carried out the genetic crosses and mouse histology. RZ, S Ali and SA performed the experiments. DAM, MDS, AJS, ZLD and RH designed, performed and analyzed viral infectivity and MusD transposition assay data. All authors read and approved the final manuscript.
